# Excess Mortality Attributed to Hurricane Irma Among Assisted Living Residents With Chronic Conditions in Florida

**DOI:** 10.1093/geroni/igab046.1104

**Published:** 2021-12-17

**Authors:** Cassandra Hua, Kali Thomas, Lindsay Peterson, Debra Dobbs, Ross Andel, David Dosa

**Affiliations:** 1 Brown University, Providence, Rhode Island, United States; 2 Brown University, Brown University/Providence, Rhode Island, United States; 3 University of South Florida, Bradenton, Florida, United States; 4 University of South Florida, School of Aging Studies, University of South Florida, Florida, United States; 5 University of South Florida, Tampa, Florida, United States

## Abstract

Little is known about the impact of hurricanes on residents in assisted living communities (ALs), especially among individuals with chronic conditions that increase their risk of death after storms. We examined how the association between exposure to Hurricane Irma in 2017 and mortality differed by select chronic conditions. With Medicare data, we identified cohorts of AL residents in 2015 (n= 30,712) and 2017 (n= 29,842 ) and compared their rates of 30-day and 90-day and mortality. We adjusted rates for demographic characteristics and other comorbidities. AL residents with diabetes were at highest risk of death after the storm; between 2015 and 2017 they experienced a 50% increase in their 30-day mortality rates (0.6% in 2015, 0.9% in 2017) and a 43% increase in their 90-day mortality rates (2.1% in 2015, 3.0% in 2017). Policy makers should consider strategies to ensure that diabetic residents maintain continuity of medical care during disasters.

